# Understanding inequities in access to diabetes technologies in children and young people with type 1 diabetes: Qualitative study of healthcare professionals' perspectives and views

**DOI:** 10.1111/dme.15486

**Published:** 2024-11-29

**Authors:** Rachel Dlugatch, David Rankin, Mark Evans, Nick Oliver, Sze May Ng, Julia Lawton

**Affiliations:** ^1^ Usher Institute, Medical School University of Edinburgh Edinburgh UK; ^2^ Institute of Metabolic Science and Department of Medicine University of Cambridge Cambridge UK; ^3^ Imperial College London UK; ^4^ Faculty of Health, Social Care and Medicine Edge Hill University Ormskirk UK; ^5^ Department of Women's and Children's Health University of Liverpool Liverpool UK; ^6^ Paediatric Department, Mersey and West Lancashire Teaching Hospitals Ormskirk UK

**Keywords:** children and young people, healthcare professionals, inequality, qualitative research, type 1 diabetes

## Abstract

**Aims:**

We explored healthcare professionals' perceptions and understandings of the factors and considerations underlying inequities in technology access in children and young people (CYP) with type 1 diabetes.

**Methods:**

We interviewed (*n* = 29) healthcare professionals working in paediatric diabetes in England recruited from (*n* = 15) purposively selected sites. We analysed data thematically.

**Results:**

Interviewees highlighted multiple, often overlapping barriers to accessing technology faced by CYP with type 1 diabetes from deprived and/or ethnic minority backgrounds. They described the impacts of deprivation on technology uptake, together with the complex social, ethnic and cultural factors that could also reinforce disparities in technology access. Interviewees further highlighted staffing shortfalls as a significant barrier to supporting CYP to use technology, especially those from under‐represented groups who they perceived as requiring more staff time to be trained to use technology. While interviewees suggested that unconscious bias has become less prominent, they reported being less likely to recommend technology (especially pumps) to CYP/caregivers who they feared would not use it safely and effectively (e.g. those with low literacy levels). Interviewees also highlighted geographical variability in the technology commissioning process (a ‘postcode lottery’).

**Conclusions:**

Our findings suggest that without targeted interventions, technology inequities may continue to persist amongst CYP from the most and least deprived areas and from white and ethnic minority groups in the United Kingdom. Additionally, our findings suggest that closing the technology gap will require large‐scale governmental and health policies aimed at fostering socioeconomic, ethnic and cultural equality alongside targeted measures to improve technology accessibility.

**What's new?**

- Inequities in access to diabetes technologies amongst children and young people (CYP) from the most and least deprived areas and from white and ethnic minority groups continue to widen in the United Kingdom.
- Despite healthcare professionals acting as technology gatekeepers, their perspectives have been underreported.
- Healthcare professionals highlighted complex, multifactorial reasons for technology access disparities, including: deprivation; social, ethnic and cultural factors; staffing shortfalls; unconscious bias; and, inequitable commissioning processes.
- Their accounts suggest that addressing inequalities in access will not be easy and that access gaps may continue to widen despite recent interventions to improve uptake amongst CYP from under‐represented groups.

## 1 INTRODUCTION

Diabetes technology, such as continuous glucose monitoring (CGM) systems, insulin pumps and hybrid closed‐loop (HCL) systems, can improve health outcomes^1^, ^2^, ^3^ and enhance quality‐of‐life.^4^, ^5^, ^6^, ^7^ However, in the UK, 2020/2021 National Paediatric Diabetes Audit (NPDA) data highlighted year‐on‐year widening gaps in technology access between children and young people (CYP) from the most and least deprived areas and between those from white and ethnic minority groups. More recently, the 2022/2023 NPDA report^8^, ^9^ has shown that, despite increased uptake in all demographic groups, disparities in technology access have worsened. These trends are mirrored in other countries^10^, ^11^, ^12^ and in adult type 1 diabetes populations.^13^

The NPDA data prompted widespread concern about inequities in technology access^14^ alongside initiatives to help address them. This included recommendations to offer real‐time CGM to all CYP from December 2022 and NHS England developing a Diabetes Treatment Technology Fund, which offered targeted funding to sites where NPDA data showed particularly large disparities in technology access. Additionally, in December 2023, the National Institute of Health and Care Excellence (NICE) published a Technology Appraisal (TA943) mandating that HCL be made freely available to all CYP (aged <18 years) with type 1 diabetes in England.^15^ Integrated Care Boards (ICBs), statutory NHS organisations responsible for providing health services and managing budgets in designated geographical areas, have been tasked with setting out five‐year plans for fulfilling the NICE TA.^16^ While these developments represent a positive step forward, it remains to be seen to whether they will close the technology access gap.

To better understand, and help address, inequities in technology access, we conducted interviews with CYP with type 1 diabetes and/or their parents/caregivers and healthcare professionals involved in CYP's treatment and care.^17^ Here, we report findings from the healthcare professionals' interviews which help illuminate reasons for the inequities in technology access observed in CYP with type 1 diabetes. The solutions healthcare professionals proposed to help address these inequities will be reported separately.

## 2 METHODS

### 2.1 Overview

We conducted in‐depth interviews informed by a topic guide, which helped ensure discussions remained relevant to addressing the study aims whilst affording flexibility for interviewees to raise issues they considered salient, including those unforeseen at the outset.^18^ The study employed an inductive approach in which data collection and analysis took place concurrently, enabling findings from early interviews to iteratively inform areas explored in later ones. Research ethics approval was obtained from the Edinburgh Medicine Research Ethics Committee, University of Edinburgh (23‐EMREC‐007, 18th April 2023). Informed consent was provided prior to all interviews.

### 2.2 Sample and recruitment

We recruited consultants/doctors, diabetes specialist nurses and dieticians who worked in paediatric diabetes care in England‐based clinics from 15 purposively selected sites identified from the 2020/2021 NPDA data^9^ as having particularly high/low uptake of diabetes technologies amongst disadvantaged groups; we also selected some sites with above average numbers of CYP from low‐income and/or minority ethnic groups.

Recruitment took place via email invitations sent by the chief investigator, and those interested in participating were asked to opt‐in directly to the qualitative research team. We also used snowballing, whereby existing interviewees were asked to send recruitment packs to colleagues who they thought might offer complementary or different perspectives to/from their own. Where possible, we recruited two healthcare professionals from each site to enhance understanding of practices and decision‐making within their team. Recruitment continued until there was a diverse sample of sites represented, good representation of different grades of staff, and no new findings were identified in new data collected.

### 2.3 Data collection and analysis

One‐to‐one interviews were conducted by RD and DR, both experienced non‐clinical qualitative researchers, between October 2023 and April 2024. The topic guide was informed by literature reviews, inputs from clinical co‐investigators, and revised in light of emerging findings and developments (e.g. announcement of TA943, which was published partway through data collection). Key topic areas explored relevant to the reporting in this article are described in Box 1. Interviews took place using MS Teams or by telephone, lasted 1–2 hours and were digitally recorded and transcribed verbatim.

BOX 1 **Main Topics Explored in Interviews (Relevant to the Analysis)**

**Table:** 

Interviewee's clinical background, current role and experience of working in paediatric diabetes care Characteristics of interviewee's diabetes centre, including number and types of staff, number of CYP with type 1 diabetes being supported, proportion of CYP from socio‐economically deprived and/or ethnic minority backgrounds Interviewee's assessment of their own diabetes centre's performance, including: number of CYP on CGM, pumps and HCL; proportion of CYP from socio‐economically deprived and/or ethnic minority backgrounds using technology; comparisons with other centres locally and nationally; how/why the centre's performance has changed in recent years Views about why the technology access gap exists nationally and locally (if relevant) Centre's experience of supporting CYP using CGM, pumps and HCL; personal experience providing such support Availability of funding, e.g. to hire staff; access to Diabetes Treatment Technology Fund Perceptions and understandings of: challenges CYP/caregivers face in everyday life and impacts of these on technology usage; particular challenges faced by CYP from deprived and/or ethnic minority backgrounds; which CYP/caregivers are more/less likely to engage with services and/or want to use technology and why (probe: social, cultural and ethnic factors) Views about: staff‐to‐patient ratios and caseload and whether this has changed over time; impact of increased technology use on workload, training needs, wellbeing and opportunities to develop rapport with CYP/caregivers Views about: the benefits of technology; who should use diabetes technology; who might 'thrive'/'struggle' on technology Experiences of recognising and challenging unconscious bias; whether/how any biases might have influenced decision‐making about encouraging CYP to use technologies Views about: limiting and enabling factors to putting more CYP on technology; the NICE TA943 and 5‐year implementation strategy, including any predicted challenges moving CYP onto HCL Experiences of commissioning and procuring technology, impacts on workload and therapeutic relationships; impacts of NICE guidance on commissioning processes and provisioning for CYP

Data were analysed by three highly experienced postdoctoral qualitative researchers (RD, JL and DR). All interview transcripts were read through repeatedly (data immersion) and cross‐compared to identify cross‐cutting themes. Deductive and inductive approaches^19^ were used to: (a) identify data that addressed our original research questions (e.g. barriers to and decision‐making about access to diabetes technologies)^17^; and, (b) capture any unanticipated and/or site‐specific issues raised by interviewees. To promote rigour and minimise risk of bias, each researcher undertook separate analyses before meeting to discuss their interpretations and reach consensus on a coding frame that captured key themes/findings. NVivo20 (QSR International, Doncaster, Australia) was used to code and retrieve data and coded datasets were subject to further analyses to allow more fine‐grained interpretations.

## 3 RESULTS

Our sample comprised 29 healthcare professionals (14 consultants, 9 nurses, 6 dieticians) with 3–32 years of experience in providing diabetes care. For further information about the sample, see Table 1. For additional quotations, see Table 2. Unique identifiers comprising each interviewee's site (i.e. 001–015) and role (i.e. C = consultant, N = diabetes specialist nurse; D = dietician) are used below to safeguard anonymity.

**TABLE 1:** Characteristics of the sample.

	*N* (%)*
Sites (*n* = 15)	
Total number of interviewees	29
Interviewees per site—range (mode)	1–3 (2)
Role	
Diabetes consultants	14 (48.2)
Diabetes specialist nurses	9 (31)
Dieticians	6 (20.7)
Years of diabetes experience	
<5 years	1 (3.4)
5–10 years	9 (31)
11–20 years	14 (48.3)
>20 years	5 (17.2)
Gender	
Women	19 (65.5)
Men	10 (34.5)
Age in years: mean, SD (range)	46.5 ± 7.9 (30–59)
Ethnicity	
Asian or Asian British	11 (37.9)
Black, Black British, Caribbean, or African	2 (6.9)
White (British, Irish, South African, Other)	15 (51.7)
Other ethnic group	1 (3.4)

* Percentages do not equal 100% due to rounding.

**TABLE 2:** Supplementary quotations.

THEMES	Additional illustrative quotations
Deprivation and socioeconomic barriers	*Lack of numeracy skills* Some families struggle with…something as simple as getting a set of scales to be able to weigh out foods and measure foods. Others will not want to sort of show that they struggle with things like maths, so they sort of go along with your teaching and your education, and you think they're understanding it, but when you try and get them to go back over it, there's a clear sort of barrier in something as simple as maths. (010_N) *Transportation difficulties* So, travel is one thing, travelling to appointments. Some of our patients, families, are quite deprived. They don't have their own transport and they often don't live on a good bus link to come to appointments. So they'll end up paying for a taxi every time…Some families or patients that are struggling more, we see them more in clinics so then that's more time off school. Again, that's more transport costs and everything. (009_D) *Impacts of digital poverty on technology knowledge* So when we have a newly diagnosed patient whose parents are educated, they can Google things. Straight away they read about pump therapy, they know exactly what it does after researching it. Then they come to us the first appointment and they ask for it. Whereas if the parents don't speak English or they're not educated and they can't do their own research, they don't ask us for it because they don't know it exists and they don't know what it is, they don't know the benefits of it, so we need to give them that education. If we don't, then they're missing out and then they end up getting technology a lot later. (009_D)
Social, ethnic and cultural factors	*Fear of technology* And if [caregivers] don't understand the technology because of their educational level or their language and understanding then it's difficult for them to gain that level of understanding to say, I have put my child's life into that little device…So there's a major fear factor. (008_N) *Cultural attitudes toward visible illness and wearable technology* I'd say what I've experienced, certainly more from an Asian background, some of the family members, they don't want it visible that their young daughter or son has type 1 diabetes because they're still in arranged marriage situations, and that often can be precluded into getting a good arranged marriage…Whether it's the caste system for people from a Hindu background or whether again from a Muslim perspective, there are certain elements within there. (007_D)
Staffing and service capacity	*Importance of home visits* And it might be that to get them on technology, they need a lot more home visits, they need a lot more support. We've got one family at the moment whose little one's just gone on to [hybrid 0:38:28] closed‐loop. He's having almost daily contact and he's having weekly home visits. So that's…there is an increase in demand on staffing time to support these ones and you have to go and meet them where they're at. (002_D) *Funding provisions for staffing* [Staffing] is expensive though and Trusts are very short of money. So it's an uphill battle. (008_N)
Unconscious bias	*Perceptions of which CYP/caregivers can ‘cope’ with technology* Technology wise we are ready to offer [it] them, provided they can handle it. But if you give the technology, like example of pump or anything, and if they don't know how to use it, I don't feel it's safe for them as well. Because, like I said, some of them struggle with doing even numbers, no literacy, numeracy skills at all. (008_C)
Postcode lottery	*Differences in ICBs and commissioning technology* Our prior approval system is really very challenging because there are certain ICBs, which approve very easily. There are certain ICBs which approve tons of the CGMs. There are some that do not approve closed loops at all. So that is a major obstacle for us. (012_C) *ICBs and NICE guidance* …it's recognised by the whole country that NICE guidance is what we should be adhering to. Problem is, it's a guidance. And it's not stipulated in law. So NICE can come out and say, all children should be eligible. And it carries a lot of weight, [but] the [ICBs] can ignore it to a certain extent, or they can put their own restrictions upon it. (008_N) *Self‐funding technology* But some parents…this sort of comes back to the difference in the socioeconomic status, so some will self‐fund certain things. So they might meet the criteria for a pump but they don't meet the criteria for CGM, so they will self‐fund CGM so they can close‐loop. Then we have other people who self‐fund both a pump and CGM. But it's a lot of money for them to self‐fund all of that. (005_C)

The findings are structured under the following themes: deprivation and socioeconomic barriers; social, ethnic and cultural factors; staffing and service capacity; unconscious bias; commissioning technology; a ‘postcode lottery’. We have not separated findings according to healthcare professionals' individual characteristics and professional roles as responses and themes cut across the dataset.

### 3.1 Deprivation and socioeconomic barriers

Interviewees recognized that many CYP and their families, especially those from deprived backgrounds, led complex lives and faced material barriers ‘whether that be money, whether that be social, family stresses’ (015_D) that prevented them from accessing healthcare services, prioritising their diabetes, and, hence, using technology:

People have very, very complex lives. People especially who are struggling…diabetes is just at the bottom of their big problems. (009_C)

As interviewees further observed, even when families tried to prioritise diabetes management, they were likely to run into practical and financial barriers. For example, interviewees noted that some pumps and HCL systems required access to smartphones, computers and the internet and, while this did not pose a problem for more affluent families, ‘it's another kind of issue for a family that may not be able to afford a phone’ (015_D).

Additionally, many interviewees reported that they had ‘a number of families that don't read or write in any language’ (015_D) and suggested that ‘a good amount of literacy [is needed] to understand what [the technology] is asking for’ (011_C). Such interviewees further observed that literacy additionally included the ability to read and write, health literacy, numeracy skills and technological competency. Interviewees suggested that inability to do basic mathematics, measure/weigh food (Table 2) and/or use phone apps (which, they said, some CYP/caregivers found too difficult) meant that some families felt less confident using technology and/or had less technology readiness. They added that, while HCL technology can automate many tasks, it still requires user input:

if you can't add up, if you're innumerate or you can't read a package to calculate the carbs, those bits of information still need to be inputted on to the machine. (007_C)

Another challenge, as interviewees observed, was unreliable and poorly linked public transportation, which resulted in families without a car or who could not afford taxi fares missing appointments (Table 2). Interviewees noted that those who missed appointments also missed opportunities to learn about, and be encouraged to use, diabetes technology. Interviewees observed how, unlike more affluent CYP/caregivers who conducted their own research at home, families who experienced digital poverty and/or had lower health literacy were less likely to know about, and hence request to use, diabetes technology (Table 2).

Finally, interviewees observed the importance of caregiver involvement in managing diabetes and using technology, especially amongst younger CYP who were still reliant on adults. Hence, interviewees noted that CYP receiving social care or where there were safeguarding issues experienced additional barriers. Interviewees added that ‘social care do not seem to understand diabetes’ (015_C) and that even if staff were trained to support CYP on diabetes technology, CYP in foster care move frequently: ‘if someone did a nationwide [study about] kids in foster care, with a social worker, how many of them have got pumps, it would tell a bad story’ (015_C).

### 3.2 Social, ethnic and cultural factors

All interviewees reported being aware of NDPA data, and, without being prompted, described how their centre fitted into the national picture, with most acknowledging that inequality persisted in their own services. Indeed, many acknowledged that families from ethnic minority backgrounds might have different needs to their white counterparts and that their centres were underequipped to fully serve these families.

In doing so, interviewees discussed language barriers, particularly for immigrant families. As they suggested, limited knowledge of English created communication difficulties both in and out of clinic, made certain technology difficult to use because educational materials and/or device settings were in English (or had limited non‐English options), and resulted in many feeling fearful and/or hesitant about using technology (Table 2). Speaking about an Ethiopian immigrant mother, (009_D) reported how:

she wants the best for her daughter, but because she can't read English, she is just terrified of having to rely on this device. She's like, *What will happen to my baby if I don't know what to do and I can't get help*?

Despite their attempts to address language barriers, interviewees reported that ‘we often can't get an interpreter in clinic’ (009_D) or experiencing difficulties finding interpreters who spoke the languages used by the CYP/caregiver. Additionally, they suggested that using interpreters could create social barriers between clinician and CYP/caregiver: ‘it's always harder to go through interpreters. It's real back and forth…and you don't have that same personal feel for what they're thinking’ (015_C). Furthermore, while interviewees at some sites reported translating their educational materials, others reported being prohibited by costs, especially at sites serving diverse ethnic minority groups speaking multiple languages.

Additionally, interviewees observed how, for some families, culturally informed ideas about illness might have prevented them from wanting to use diabetes technology (Table 2). As they noted, some families ‘try to hide the diagnosis from the rest of their community’ (012_C), which could make wearable technology ‘not acceptable because it's a visible sign of disease’ (007_C).

Similarly, some suggested that adolescents often disliked having anything attached to their bodies, which could mark them as different from their peers. While interviewees acknowledged this as being an issue regardless of cultural or ethnic background, several observed that, ‘because a lot of the technology is white, and a lot of our families do not have white skin, it's more obvious’ (015_D).

### 3.3 Staffing and service capacity

Excepting a minority who felt ‘very lucky to have a lot of staff’ (001_C), most interviewees felt that staffing shortfalls limited the number of CYP they could start and support using diabetes technology: ‘If you want to develop your service and you want to give the kids and the families the kind of level of care they require now…with technology developing at such an incredible rate, we need to have more staffing’ (008_N). While interviewees at some sites described having expanded their teams, most noted that these had ‘not expanded as much as our patients have’ (010_C).

Low staffing was reported as having had multiple ripple effects, often exacerbating the disadvantages facing those from deprived backgrounds. Because ‘the NHS is always gonna be pushed to see more people in less time’ (015_D), interviewees often described how they felt it had been necessary to make judgement calls about who should be moved onto diabetes technology first. In doing so, some noted having prioritised more educated, affluent families who required less training and input from staff over CYP/parents who they perceived as requiring more time (e.g. non‐English speakers or those with lower numeracy skills):

…because you [can] put a white, English‐speaking, middle‐class family who will get it on [a HCL] in two hours and it's going to take you eight hours to do it with a family who don't speak the language…because they've [not] got literacy skills, you think, *Well, I could do four patients versus one patient*. (010_C)

Interviewees additionally observed how staffing shortages had led to home visits becoming less frequent, further affecting access to diabetes technology. As several pointed out, while home visits placed further demands on staffing time, they were essential for supporting CYP/caregivers with additional needs to use technology (Table 2). Interviewees observed how developing rapport with families, especially those from deprived and/or ethnic minority backgrounds, helped with understanding and addressing their concerns as well as building their confidence and, hence, encouraging technology use:

We need to see [the child] at home and we need to encourage them about pump therapy, find out what the barriers are, because just coming to clinic doesn't work. They don't open up in clinic very well, especially if they have that communication barrier. (009_D)

All interviewees reported examples of burnout, even those from better‐staffed sites. As 008_N explained, caseloads had expanded due to an increased average intake of new CYP at diagnosis because ‘Covid has nationally been seen as a trigger mechanism’ for type 1 diabetes. While most reported wanting to hire additional staff, including technology support workers to perform non‐clinical tasks supporting technology use, they also described having been informed that no funding was available (Table 2).

While some noted that NHS England had encouraged sites to improve their technology usage, most suggested their motivation to do so had been borne from awareness of NPDA data, improved availability of technology, and because ‘[w]e want to give [our CYP] the best quality of life’ (003_N). However, increasing technology usage was described as having come at a cost, with interviewees noting that finding time for training to keep up‐to‐date with new technology was ‘hard’ and that ‘a lot of us do end up doing it outside of working time’ (015_D). Additionally, they highlighted a consequent risk of burnout, with 012_N reporting how a colleague had gone on extended sick leave ‘because she was knackered. She's burnt out’.

For these reasons, many interviewees felt that staffing shortfalls would be the limiting factor in moving a large number of CYP onto HCL quickly, even with the NICE TA943: ‘It's a common team opinion that the resources, current resources, will not be enough’ (012_C). Furthermore, interviewees noted how putting CYP onto pumps is time consuming and, hence, suggested that it would be quicker to initiate HCL use in those already on pumps. However, as some further noted, this would mean that, in practice, more white and/or affluent CYP (already on pumps) would get earlier access to HCL: ‘we've got a cohort of patients that are on manual pump, but we're hopefully moving them across to closed loop [imminently]’ (003_N).

### 3.4 Unconscious bias

Despite a consensus that unconscious bias ‘is significantly less…compared to what it was twenty years ago’ (006_C) interviewees acknowledged that bias still plays a role in inequities in access to diabetes technology:

[When] a patient's family walk[s] in, we're making judgements…Are they educated?…How they speak, what they wear…[A]s healthcare professionals…it's always easier to connect with the middle‐class family who will want to do everything for the child, is educated, who talks like you…I think we would be lying to ourselves if we said that doesn't exist. (010_C)

Interviewees indicated that, when possible (e.g. in well‐staffed/resourced sites), they tried to offer technology to everyone. However, they also suggested that some CYP/caregivers (e.g. with low literacy levels, from single‐parent families, or those perceived to be ‘unengaged’ and/or belonging to ‘chaotic’ families) would be less likely to ‘cope’ with technology and use it safely (Table 2). While interviewees noted that not recommending technology to these CYP could be considered problematic, they also described how their decisions were usually ‘borne out of good reasons’ (015_C):

We would be worried about somebody, if someone wasn't managing their diabetes well, and it was sort of at breaking point, we'd think, well if we put them on a pump we could potentially increase their risk and tip them into DKA sooner. (010_N)

Finally, some interviewees acknowledged ‘a positive kind of bias towards those families who are aggressive’ (012_C) in asking for technology, usually ‘higher status…patients and parents who read about these things in the news…and in ethnicity white British’ (012_C).

### 3.5 Commissioning technology: a ‘postcode lottery’

Interviewees often described the process of commissioning and procuring technology as varying across different geographical regions (what many termed a ‘postcode lottery’) wherein one diabetes centre could commission technology through their local ICB and ‘never [be] questioned for how many pumps, how many CGMs we use’ (015_D), while a neighbouring centre served by a different ICB could see many of their requests rejected (Table 2). Interviewees also reported applications for technology being turned down even when CYP met NICE criteria (Table 2).

Escalating the situation, often in the form of appeals, was described as ‘really time‐consuming and frustrating’ (012_N) for already burnt‐out staff. Additionally, interviewees described how rejected applications could damage therapeutic relationships with those already struggling with self‐management and attending clinic: ‘that is a big challenge to explain to the parents as to why the other family has got all they need and why they don't have it just in a kilometre geographical difference’ (012_C). Some interviewees further added that, while some parents (typically those who were more affluent) resorted to self‐funding in such situations, for many this was prohibitively expensive (Table 2).

## 4 DISCUSSION

In this article, we have reported healthcare professionals' perceptions and understandings of the factors and considerations underlying inequities in access to technology amongst CYP with type 1 diabetes from deprived and/or ethnic minority backgrounds. Echoing studies from the United States,^20^, ^21^ our research suggests that the social determinants of health, the nonmedical factors that can influence health outcomes,^22^ can play a significant role in promoting and exacerbating technology inequities (specifically: economic and/or familial (in)stability, education, literacy, access to digital devices and connectivity, transportation and sociocultural inclusion). Additionally, our findings indicate that provider bias, in conjunction with contextual factors, such as understaffed diabetes centres and inequitable commissioning processes (i.e. the ‘postcode lottery’ which others have also highlighted^23^), can compound pre‐existing inequities. In doing so, and alongside studies reporting CYP's perspectives,^24^ we have shown that the factors underlying inequities in access are complex, multifactorial and sometimes highly pernicious. Hence, tackling these inequalities is unlikely to be a simple and easy task.

In December 2023, NHS England announced a five‐year implementation strategy for the NICE TA943, which will make HCL technology freely available to all CYP with type 1 diabetes in England.^16^ Given that NICE TAs are legally binding,^25^ the TA should, in theory, address the differential referral and commissioning processes reported in this article. However, our findings raise important questions about whether technology disparities might initially be exacerbated by the TA's launch, especially in the transitional years while ICBs develop and deliver their five‐year action plans to promote HCL access. Indeed, interviewees' accounts suggested that, if staff‐to‐patient ratios are not increased or staff workloads not improved by other means, healthcare professionals might initially prioritise CYP who they perceive as requiring less time and effort to move onto HCL. In practice, this will likely mean those who are affluent, educated and English speaking, and/or already using pumps (a group which are also more likely to be affluent, educated and white^8^, ^9^). For this reason, as others have argued, our study highlights the need to improve funding and provisions for staffing.^26^ Increased staffing could also facilitate rapport and trust building, a lack of which, as others have shown,^27^ can act as technology barriers for Black CYP/caregivers in particular. Additionally, increased staffing could allow more home visits, which our interviewees cited as being important for encouraging technology uptake amongst under‐represented populations.

In addition, our findings suggest that unconscious bias, which has also been identified by others,^28^, ^29^, ^30^, ^31^ must be addressed to help tackle technology inequities fully. As others have argued, if left unchallenged, provider bias may continue to lead to information gatekeeping,^32^ damage provider‐patient relationships,^33^, ^34^ and, as our study has shown, it may also influence which CYP/caregivers healthcare professionals decide to spend their (limited) time educating and supporting, all of which could influence technology uptake. While unlikely to be sufficient on its own,^35^, ^36^ unconscious bias training, including presentations of case studies involving CYP healthcare professionals would expect to struggle doing well on technology, could help challenge healthcare professionals' notions of ‘ideal’ candidates for technology.^30^ However, unconscious bias training may be more immediately effective in well‐resourced sites where staff have time for additional training and are not pressed to make pragmatic, hard‐nosed decisions about who to prioritise for technology (e.g. CYP they feel will require less time and fewer resources). Again, this points to the urgency of increasing provisions for staffing, since, as our findings suggest, staffing shortfalls create conditions that promote biases and inequitable care.

Consistent with others,^21^, ^33^, ^36^ our study suggests that technology disparities will continue to be exacerbated by the social determinants of health without targeted interventions. Such interventions could include additional education and training, so that CYP/caregivers have the requisite numeracy and literacy skills to use technology safely and confidently. We also recommend improving accessibility for non‐English speakers by translating educational materials and ensuring devices have sufficient language options─this might be facilitated by enlisting the help and support of industrial partners and manufacturers. More reliable and affordable transportation could enable CYP/caregivers from low‐income groups to attend appointments and learn about technology in the first place, while an improved social care system could provide CYP with caregiver consistency and help with technology use. Peer support could also help improve education and understanding and reduce stigma around visible illness and technology.^37^ Given that technology can promote or undermine certain values, as others have argued,^38^ companies could move toward making technology in more inclusive shades, as we have seen with bandages and ballet shoes.^39^ In the meantime, ensuring that skin‐toned patches to cover sensors and pumps are made freely available could act as a stopgap.

### 4.1 Strengths and limitations

A key strength was our qualitative design, which allowed healthcare professionals' perspectives and experiences to be probed and explored in‐depth, together with our ability to adapt interview questions to accommodate the rapidly changing diabetes landscape. Another strength was the involvement of three experienced qualitative researchers in data analysis as this helped reduce potential researcher bias; we also involved clinical co‐authors in interpretating the findings and considering their clinical implications. We were successful in recruiting healthcare professionals from a diversity of sites, which contributed to the richness of our data. However, because we used an opt‐in approach, our interviewees may have held particularly strong views. Healthcare is devolved in the UK and our study was restricted to England; hence, some findings may not be generalisable to UK as a whole. Further research could be conducted in other parts of the UK and in other countries where systems of healthcare delivery may be different and where access to technology is more limited or funded by private health insurance schemes. Finally, our study focused on paediatric populations, but it is likely that many of the barriers we have identified will be applicable to adult populations; indeed, arguably some barriers might be exacerbated by lower staff‐to‐patient ratios in adult type 1 diabetes clinics. Future research could explore barriers to technology access in adult populations.

## 5 CONCLUSION

While healthcare professionals, such as those who took part in our study, appeared highly motivated to address technology access inequities, individual professionals cannot bear the responsibility of redressing systemic failures alone. Instead, large‐scale governmental and health policies aimed at fostering socioeconomic, ethnic and cultural equality alongside targeted measures to improve technology accessibility will be essential for bridging the technology access divide. Paying attention to underlying social determinants of health, as well as staffing levels and provider bias, will likely be key to any policies or strategies for mitigating inequities in technology usage.

## AUTHOR CONTRIBUTIONS

J.L. conceived and designed this interview study with healthcare professionals with input from M.N. who conceived and designed the wider UNBIASED study. R.D. and D.R. collected the data, which was then analysed by R.D., D.R. and J.L. R.D. drafted the manuscript with input from J.L. and D.R. All authors reviewed, edited and approved the final version.

## FUNDING INFORMATION

Understanding Inequalities and Barriers to Accessing Diabetes Technology in Children and Young People with Type 1 Diabetes study is funded by Diabetes UK (Grant reference number: 22/0006434) with Prof Sze May Ng, OBE as Chief Investigator/grant holder and Prof Julia Lawton, Prof Mark Evens and Prof Nick Oliver as co‐investigators. The views expressed are those of the author(s) and not necessarily those of the funding body. The University of Cambridge has received salary support for MLE through the National Health Service in the East of England through the Clinical Academic Reserve.

## CONFLICT OF INTEREST STATEMENT

M.N. declares honorarium from Sanofi and Insulet. M.E. has been a member of advisory panels and/or received speakers fees from NovoNordisk, Eli Lilly, Abbott Diabetes Care, Medtronic, Dexcom, Ypsomed, Pila Pharma and Zucara. N.O. reports grants paid to their institution from National Institute for Health and Care Research, Diabetes UK, Helmsley Trust, Dexcom and Medtronic Diabetes; speaker fees from Tandem Diabetes, Sanofi, Dexcom, Astra Zeneca and Medtronic Diabetes; and participation on advisory board for Medtronic Diabetes and Roche Diabetes. R.D., J.L. and D.R. have no conflict of interests to declare.

## CONSENT

All research participants provided written informed consent including for anonymized information to be published in this article.

## Data Availability

The datasets generated and analysed for this study are not publicly available due to risks to individual privacy. However, they are available, via the corresponding author, on reasonable request.
